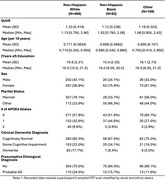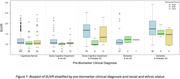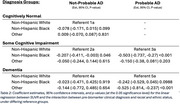# Discordance in the presumptive etiologic diagnosis and amyloid burden by ethnoracial status

**DOI:** 10.1002/alz70856_107729

**Published:** 2026-01-09

**Authors:** Paul R Gaona‐Partida, Christina M Magana‐Ramirez, Josh D Grill, Daniel L Gillen

**Affiliations:** ^1^ University of California, Irvine, Irvine, CA, USA

## Abstract

**Background:**

In early and preclinical Alzheimer's disease AD clinical trials, amyloid biomarker criteria have resulted in differential ineligibility among racial and ethnic groups. We sought to quantify differences in PET‐derived amyloid levels across racial and ethnic groups of similar clinical diagnosis and presumed disease etiology.

**Method:**

We utilized data from the Standardized Centralized Alzheimer's and Related Dementias Neuroimaging (SCAN), a newly implemented initiative funded by the National Institute on Aging in conjunction with the National Alzheimer's Coordinating Center (NACC). Our primary outcome was PET‐derived amyloid burden using standardized uptake value ratios (SUVRs) shared through the SCAN initiative. Racial and ethnic status were categorized as Non‐Hispanic Black (NHB), Non‐Hispanic White (NHW), and the remaining participants collated into together (Other), using data obtained from NACC's Uniform Data Set (UDS). We used ordinary least‐squares regression to model differences in mean amyloid levels across racial and ethnic groups, with stratification by clinical diagnosis and presumed disease etiology while adjusting for potential confounding factors.

**Result:**

We analyzed data on *N* = 661 NACC participants that had corresponding amyloid PET data available. Table 1 provides basic demographics of the study sample. Figure 1 depicts the distribution of amyloid levels by racial and ethnic group, clinical diagnosis, and presumptive etiology. Table 2 yields the estimated mean differences in SUVR levels by racial and ethnic group stratified by clinical diagnosis and presumptive etiology. After adjustment for potential confounding factors we estimated that NHB participants with cognitive impairment (impaired not MCI or MCI) and probable AD had mean SUVR ‐0.503 lower than comparable NHW participants (95% CI: (‐0.737, ‐0.270); *p*‐value: <0.001). Among participants with a dementia diagnosis and probable AD the estimated mean difference was ‐0.242 (95% CI (‐0.529,0.045); *p*‐value: 0.099) comparing NHB to NHW.

**Conclusion:**

These results add to a growing literature examining amyloid biomarkers across racial and ethnic groups. A limitation of this study is the small sample sizes in several of the groups analyzed. Further research is needed to assess these observed potential differences and the implications to AD trial eligibility and treatment.